# Lectures on Yellow Fever, Its Causes, Pathology, and Treatment

**Published:** 1848-06

**Authors:** 


### BIBLIOGRAPHICAL NOTICES.

*Lectures on Yellow Fever, its Causes, Pathology, and Treatment.* By JOHN HASTINGS, M. D., United States Navy. 8vo. pp. 69. Philadelphia: Lindsay & Blakiston. 1848.

Although we have had so much lamentable experience of yellow fever in the Southern portions of the Union; and the medical officers of the army and navy, both of this and other countries, have constantly been engaged, in certain latitudes, in observing and combating it, there is no disease whose etiology and pathology, and, in a certain degree, treatment, are more unsettled. Not long since, in noticing the appearance of another part of the valuable Cyclopædia of Dr. Copland, we referred in a pointed manner to the *quæstio vexata* of its communicability or non-communicability —a question as undecided as ever, and not, we think, set at rest by the testimony of the author of the work before us, notwithstanding that his impression is very decided, that it cannot be conveyed by one person to another.

The author of these "Lectures"—a member of that valuable corps, which has been and is an ornament and a credit to the profession—is one of those who was lately thrown in the midst of yellow fever. Yielding to the solicitation of his medical friends, on his return he concluded to prepare a paper on the disease whose devastations he had witnessed, and to which he had himself nearly fallen a victim. Perhaps in no case have the zeal and devotion to duty which were exhibited by him and some of his brother officers during the epidemic of last summer in the Gulf of Mexico, when sickness and death surrounded them on all sides, been exceeded. At the naval hospital on the island of Salmadina, the late Dr. Howard Smith and himself treated upwards of *four hundred cases*, and with great success. It was in this place that the veteran Dr. Kearney, who had volunteered his valuable services in the Gulf, breathed his last; and that Drs. Howard Smith and Bates were struck down in the active and fearless discharge of their duties. Owing, indeed, to the death and sickness of nearly all the medical officers on the station, professional aid was not always at hand.

In the course of the whole epidemic in the Gulf, Dr. Hastings was engaged in the management of nearly, or quite, twelve hundred cases. He is surely, therefore, entitled to speak, and to be heard with respectful attention; and it was a merited compliment on the part of his auditors in Philadelphia to request that he should furnish them with a copy of his "Lectures" for publication.

Dr. Hastings admits, that the disease rests in much obscurity; but he is of opinion that there is no difficulty inherent in the subject, and that it is "owing to our want of research and investigation, and too great yielding of opinion to European authorities."

To a certain extent this may be true. Observers, however, in the Southern portions of the Union have been, and are, engaged in investigating its pathology, and it can scarcely be conceded, that the ideas of M. Louis "are made the constant criteria of this disease, from the fact that he once saw it," seeing that the testimony from these recent investigations, here and elsewhere, is somewhat unfavourable to his examinations being regarded as universally, or even generally admitted. M. Louis, himself, was especially careful that his descriptions should be considered as not applying to *all* epidemics of yellow fever. With the propriety and candor that distinguish him, he begs the reader of his work on the yellow fever of Gibraltar to remember, that it is not a treatise on yellow fever, but a history of *the epidemic yellow fever which prevailed in Gibraltar in* 1828. "All the general facts"—he states emphatically,—"which result from my analysis, may not be found in other epidemics."

We cannot follow the author through all the topics which he has discussed, but must confine ourselves to those that are the most important, touching upon them in the sequence adopted by him.

His views on the etiology of the disease are not calculated, we think, to satisfy the good generalizer. "I feel perfectly satisfied," he says, "in my own mind, that there is but one cause capable of exciting this disease; and that cause is to be found in the malaria, or exhalations from alluvial or marshy soil, and that too, from marshes subject to periodic inundation and draining. The period at which the poison is eliminated is after the draining of extensive marshes, and during the process of desiccation. Close attention to the subject, and well directed observation, make this clear to the mind, wherever the disease is found in the endemic form, and it is only met with in such situations and at such times. This is clearly proven by the condition of things in the lower portion and marshy districts of Florida, where the disease is endemic, and often prevails to a frightful extent, and where it was my fate to see it in its worst form.

"Now here we have the two conditions of soil brought in immediate contrast, viz.: That constantly inundated, and that subject to periodic inundation and draining. And whilst the disease is frequently raging in one situation it is never known in the other. Thus, when the men were operating in the everglades of Florida, during the late Seminole war, they were never attacked by the disease, whilst those kept outside upon the coast, and in the mouths of rivers, were sickening and dying of the fever. The everglades are low marshy districts, having a coral base, deeply covered by decayed vegetable matter, and always covered by fresh water, being the sources of several rivers emptying into the sea. Upon approaching the coast, however, we find these marshes subject to periodic overflowing and draining. And here alone we have the disease, and that too after the rainy season. This, I believe, is also the case in every part of the West Indies where the disease prevails. And this was found to be true in Mexico in the epidemic of last year: here, also, the disease is confined to the same condition of soil, for it is not found upon the elevated and dry lands, but always confined to the districts of drying marshes." p. 23.

It is evident from the above, that the author considers the source of yellow fever as of intermittents and remittents to be paludal; and if any doubt existed on this subject, it would be removed by the following assertion:

"Intermittent fever, I believe, is universally admitted to be the result of marsh miasmata. Now it is positively certain, that where yellow fever prevails, there also is every variety of intermittent, and very generally all varieties of remittent and bilious fevers: these diseases arising from the same cause by different degrees of intensity of the poison. The cause of the varying qualities of this principle have never yet been explained, or fully comprehended, although its effects are constantly observed." p. 24.

We are not by any means satisfied with these sentiments, which seem to us to be suggested by a too restricted view of the subject. That the disease is an endemico-epidemic, or, in other words, caused by a union of local emanations with a favouring condition of atmosphere, can scarcely admit of question. Yet of the precise nature of these conditions we are in as much darkness as we are in regard to those that give occasion to fever unquestionably paludal.

Yellow fever prevails extensively where no marshes exist; whilst intermittents and remittents are fearfully rife where there is no yellow fever. Lamentable experience appears to teach us, that in certain regions, under the influence of elevated temperatures, localities exist that induce yellow fever; and yet in these very localities we often find all our speculations set at nought. We were much struck with an array of facts presented in a southern contemporary a few years ago, strongly illustrative of our ignorance on this subject. "The health of New Orleans,"—says the Editor of the *New Orleans Medical Journal* in referring to the deaths of that city in the summer and autumn of 1844—"was perhaps never known to be better than from the beginning of this year up to the present time. No epidemic whatever has prevailed; and the most extensive practitioners of the place unite in the testimony, that they have never had less to do during the same period. The summer has been the hottest ever experienced, with frequent showers during July and August. Thus, it would appear, we have had a large share of *two* of what have generally been considered the most *essential agents* in the production of the remote cause of summer and autumnal diseases; i. e., *heat* and *moisture.* As to the other ingredients, viz., *dead vegetable* and *animal matter,* one would suppose there never was any deficiency about such a place as New Orleans. Well, we have here all the *hypothetical elements* of *hypothetical malaria*—but where are the much dreaded consequences? We will go on with our statement of facts, and our readers may draw their own conclusions. The Mississippi, which runs along the borders of our city for about three miles, has probably not been higher within *fifty years* past.

Its waters, being considerably above the level of the city, were permitted, by means of culverts, to pass into the gutters, and thus to flow along the principal streets in continued streams to the swamps in the rear. These lively streams, to the width of at least a mile along the heart of the city, have continued to flow from May to about the 16th of September, when their source was cut off by the falling of the river. The descent from the river to the swamp is about four or five feet; lessening as you recede till the waters in the gutters at the back of the city (a distance of a half to three quarters of a mile) are almost stagnant. The scavengers usually draw out the thick muddy contents with hoes, and after drying it is carted off. The supply of fresh water this season must have had a salutary influence upon these *filthy sewers*. Since the decline of the river, an immense *batture* along the whole extent of the city has been exposed to the rays of an autumnal sun, but little mitigated by cloud or rain for four or five weeks past. The effluvia from this batture are quite offensive, both in the morning and evening. Such is the state of the case; and under such influences New Orleans has been, and continues to be, one of the healthiest cities in the world."

We are not justified, it seems to us, in affirming more—in our existing state of knowledge or of ignorance, whichsoever it may be termed—than that yellow fever is a regional or local disease; but what is the condition of the locality that occasions it, we know not; or what precise atmospheric condition is demanded that may render the local causes effective in the development of the disease. Our blindness on this point is like that of old Gobbo—more than "sand:" it is "high gravel." Much consideration has, however, induced us to disbelieve in the fancied agency of marsh-poison in its causation, and, therefore, to separate the consideration of *continued* yellow fever from that of the *intermittent* and *remittent* forms. Still, we cannot say, that they who refer them all, as well as typhus and typhoid fever, to some form of malaria, are positively wrong. We wait for developments; and whether the blindness in which we are placed is to be an opacity of a temporary character that may admit of extraction, or be of the permanently amaurotic cast, time must determine.

Dr. Hastings affirms, that "he has never seen a single instance where there was the least cause to suppose that the fever originated on board ships"—notwithstanding "he has frequently seen ships in the conditions said to give rise to it;" and he "never saw it on board any of them unless it was prevailing on shore." But then—it must be observed—he is of opinion, that when it does originate in ships, it is owing to malaria being conveyed to them from the land; and in order to account for the disease occurring on shipboard at a great distance from the shore, he is, of course, compelled to admit that it can be conveyed to very great distances.

"We can readily," he says, "comprehend this, when we take into consideration to what immense distances from any land infusoria have been collected upon the decks of vessels; for instance, ships running parallel to the coast of Africa, at the distance of one, two, and even three hundred miles from the coast, have had sufficient numbers of these small animals found upon their decks to furnish demonstrations of many varieties of animalcules." [Dr. Hastings obviously refers here to the fossil infusoria found in the particles of earth falling on the sails and decks.] "Also minute fragments of stone, and various earths have lodged upon the sails of vessels, hundreds of miles from land; and is it not therefore reasonable to infer, that material marsh effluvia can be, and are conveyed to great distances through the medium of the air? I have seen this take place, if it be possible to trace cause from effect. In the past summer there were a great many naval and merchant vessels lying at the anchorage at Anton Lizardo, (twelve miles south of Vera Cruz,) at the distance at least of three miles from the shore, with which there was no communication, and yet men were constantly attacked by the fever, notwithstanding they had not left their ships for a long time before the disease appeared. Again, upon the Island of Salmadina, where the naval hospital was placed, (at Anton Lizardo also,) but removed not less than five miles from the Mexican shore, being a small coral island, perfectly dry and very healthy. Yet upon this spot there were men attacked by the fever who had not been off it for weeks and months." p. 27.

In order to admit, however, the deductions of the author, we must conceive that malaria is more tangible—more grossly material—than has been generally imagined. The conveyance of "blocks"—as they have been termed by one able naturalist—a thousandth part of an inch in diameter, for some hundred of miles from land, assuredly facilitates our comprehension of the possible mode of conveyance of animalcules or fungi to immense distances, and gives countenance to the possible production of disease in this manner; but it is difficult for us to suppose, that they could be wafted by the winds to such distances without being so diffused in the air as to render them innocuous. But when we admit, the *possibility* of disease being thus induced, the *onus probandi* must rest on those who affirm that it is so, and the *onus* is sufficiently heavy. Under the idea, that malaria is essentially gazeous, it would be impossible for us to imagine, for a moment, that it could be transported in a sufficient state of concentration to the distance mentioned by Dr. Hastings, and be still capable of engendering endemic disease. In the case of the malaria, which gives occasion to intermittent fever, it has been sufficiently shown, that it can only act with the necessary virulence within a certain distance, which will of course vary according as the emanations are more or less virulent. It was long ago observed by Sir Gilbert Blane, that not only the crews of the ships in the road of Flushing were entirely free from the endemic of Walcheren, but also the guardships, which were stationed in the narrow channel between Flushing and Beveland, the width of which is about 6000 feet; and although some of the ships lay much nearer to one shore than to the other, there was no instance of any of the men or officers being taken ill with the same disorder as that with which the troops on shore were affected; whilst ships at the distance of 3000 feet, and even farther, from swampy shores in the West Indies, have been affected by the noxious exhalations; and the same thing has been observed in the India ships in the channel leading to Calcutta. Baron Humboldt long ago observed, that the farm of Encero, situated above Vera Cruz, is a stranger to the insalubrity which reigns over the whole coast. The elevation of the farm is 3,095 feet, and it forms, according to him, the highest limit of the yellow fever. In the neighbourhood of Rome, M. Rigaud de l'Isle endeavoured, by some observations, to fix the point at which the malaria is innoxious. This he considers to vary from 682 to 1000 feet above the level whence it emanates.

Dr. Hastings is not, however, the only individual who has believed in the ready transportation to, and activity of malaria at, great distances. The late Dr. McCulloch thought that the east wind has the power of thus conveying it; and he had little doubt—so he wrote—that whenever malarious disease prevailed in Edinburgh, the poison was transported from Holland! The *nugæ*

*canoræ* to which he gave utterance on this subject almost surpassed belief.

But we have dwelt too long, for our restricted space, on the etiology of miasmatic diseases; and can only add, that Dr. Hastings infers, that "there seems to be no reasonable ground for the belief of contagion" in yellow fever.

The semeiology is carefully, and, we doubt not, correctly given. The disease in fatal cases, "where death has not been anticipated by any accidental condition of the system, such as disease of the heart, large arteries, or strong predisposition to apoplexy, terminated—the author says—"almost universally" on the seventh day. "Yet there are some cases that do not terminate fatally for ten days, two weeks or more."

He has never seen a case recover where undoubted hiccough had set in; nor one after positive black vomit had occurred.

In regard to the pathology of yellow fever, he expresses himself in some detail. In all "pure cases," by which we understand him to mean those that terminate fatally on the seventh day, the following organs were found affected, and no others; and the pathological changes were precisely alike in all. The brain and spinal marrow were greatly congested and hardened: he found, likewise, congestion, softening and sphacelus of the mucous membrane of the stomach, with the removal or loss of part of its surface; and contraction and cartilaginous hardness of the liver. "These comprise all the changes effected by a fatal attack of yellow fever terminating on the seventh day."

In regard to the colour of the liver, Dr. Hastings found it to resemble very much that of old boxwood.

When the disease terminated fatally at a later period, as on the fourteenth day, or subsequently, very different pathological appearances presented themselves.

"In these cases, the brain and spinal marrow are softened; their membranes are thickened, and there is generally a very large amount of yellowish or bloody serum within the cranium and spinal canal. The heart and lungs are healthy. The stomach is in much the same condition as already described. But the mucous membrane of the duodenum, small and large intestines, is greatly injected, thickened, and softened; of very dark colour, and in some cases removed or destroyed in patches. The glands of Peyer and Brunner are injected and enlarged. The whole tract of the mucous membrane resembling the change of structure met with in patients dying of typhus fever, but not to the same extent. The stomach is sometimes filled with black vomit, and the intestines contain dark matter resembling it. The mucous membrane of the bladder is injected with blood, and spotted with many scarlet puncta. The spleen, if altered at all, is rather softer than natural; but in cases where the subject had laboured under intermittent fever of long standing, it was invariably of large size and very soft. The liver is engorged with dark blood; about natural size, of dark colour and softened. Thus showing at this advanced or protracted stage of the disease, a complete breaking down of the structure of the brain, spinal marrow, liver, and mucous coat of the stomach and intestines. The skin upon the thighs, arms, abdomen and breast is often covered with petechiæ. The albuginea and the whole surface of the body is much darker in colour than those dying at an earlier period. This is observable even before death takes place. The lips, tongue and gums are covered with a dry brown crust, the tip and edges of tongue red, and almost or quite raw. There is little or no emaciation of body. And I have never seen in any case the least reason to suspect any change in the dimensions either of the stomach or intestines. The kidneys and other viscera are found to be in normal condition. The blood remains in its liquid state in those who die; but there does not appear to be any peculiarity in that taken from yellow fever patients. It is observed to be cupped, has the buffy coat and same appearances as all blood taken from those suffering by inflammation and a high state of fever. It contains, doubtless, a large amount of bile or its constituents, owing to the crippled or suspended functions of the liver." p. 45.

The true composition of black vomit, "beyond all reasonable doubt"—says the author—"is pure blood mixed or combined with hydrochloric or acetic acid." In this he accords with Drs. Nott and Lewis of Mobile, who state, that in the epidemics of 1843 and 1844, the black vomit was acid, and that a correct idea of it may be formed by treating blood with diluted chlorohydric acid, and adding to this a little gum water or flaxseed tea, to represent the mucus of the stomach.

After a careful consideration of the manifestations of the disease, together with the pathological phenomena, Dr. Hastings is fully of opinion, that the immediate or most active cause of death must be looked for in the effects upon the brain; "since the diseased condition of the liver and stomach would not destroy life in so short a time, although they certainly assist the brain and its appendages very much, in hastening its final conclusion."

We have no doubt, that yellow fever is essentially a blood disease; and that the different organic changes are the result of the modifications of a fluid which furnishes the pabulum for every act of secretion and nutrition; and it is *probable*, that death may be more immediately connected with the material condition of the nervous system than with that of the liver and stomach. From the appearances in the yellow fever of Gibraltar of 1828, M. Louis was wholly at a loss to account for death. Dr. Hastings, after citing this, observes, that "he" (M. Louis) "never enquired into the condition of the brain and spinal marrow, but passes them by without a word, and even without observation." p. 47. In this Dr. Hastings is in error. M. Louis does describe the lesions observed in the brain and spinal marrow; but he considers them trivial, and not peculiar to the disease. In the extent and nature of the lesions of those parts, there is certainly great difference in the observations of these two gentlemen—each, doubtless, accurately depicting what he himself had observed in the epidemic that fell under his notice.

Lastly, as regards the treatment—a point which has been the subject of much dispute at all periods, but on which the opinion of the author is positively, it may be, too dogmatically expressed. "It seems impossible"—he says—"that there could exist but one [more than one?] opinion "upon the subject; it is as clear as the noonday sun;" and again—"Can there be but one [more than one?] proper mode of managing such a disease? Certainly not." Regarding the disease as one of "high inflammatory power of the general system," he knows of no means "so likely to respond to our desires as the lancet, calomel, opium or its preparations, counter-irritants, saline purgatives, and stimulating enemata"—the old treatment, by the way, so strongly urged by Drs. Rush, James Johnson and others, and pursued by the British practitioners in the Yellow Fever of Gibraltar of 1828, but apparently with less success than the milder methods employed by the Spanish physicians.

Under his method of treatment, Dr. Hastings lost only one-half per cent. of those received in the early stages of the attack, certainly a very small proportion. We have no means, however, of instituting a statistical comparison between the results of this and other methods of treatment; and we recollect some months ago having seen a statement from one of the surgeons of the army very much in accordance with the concluding remark of Dr. Hastings—that "there is no disease more entirely under the control of medical treatment than yellow fever." Was the treatment adopted by that medical officer—we allude to Dr. Barton—identical with that used by Dr. Hastings? In a disease which runs its course so rapidly—in all diseases that run their course so rapidly—no matter whether highly inflammatory or not; and where alterations of nutrition supervene so speedily under modifications of the fluid which at the same time bathes and nourishes the different tissues, the sooner a new action is excited in the system by mercurials the better. The supervention of that new action is generally aided by the abstraction of blood; but it would be interesting to know what would be the effect of the employment of full sedative doses of opium, along with mercury, pushed so as to touch the mouth if possible, from the very onset of the disease, without the abstraction of blood. We should be glad, indeed, to have the results of every form of treatment, from the expectant to the most active, placed before us in statistical detail, before we can make up our mind as to that which is really the most efficacious;—these results deduced from the treatment of the same epidemic, and under circumstances of locality, accommodations and attention as nearly identical as possible. In the meanwhile we must repeat, that the cipher of mortality under the treatment pursued by Dr. Hastings is exceedingly low.

We have bestowed unusual space to a notice of a work consisting of only sixty-nine pages. It is on a subject so interesting, however, and the author is in all respects so estimable, that we could not well pass it by with a shorter notice. For the details we must refer to the "Lectures" themselves, and conclude with the synopsis given by Dr. Hastings of the Pathology, Symptoms and Treatment of the disease.

"*Symptoms.*—Rigor; fever; injected eyes; pain in head, small of back and lower extremities; tenderness of epigastrium; emesis and black vomit.

"*Treatment.*—Bleeding; purgation; ptyalism; and counter-irritation.

"*Pathology.*—Thickening of the membranes of brain and spinal marrow, and hardening of their substances; almost cartilaginous firmness of liver, with discoloration; thickening and sphacelus of mucous membrane of the stomach. These changes consequent upon meningitis, hepatitis and gastritis." p. 69.

